# Influence of different drilling protocols and bone density on the insertion torque of dental implants

**DOI:** 10.4317/medoral.25804

**Published:** 2023-06-18

**Authors:** Ana Fernández-Olavarria, Aida Gutiérrez-Corrales, Maribel González-Martín, Daniel Torres-Lagares, Eusebio Torres-Carranza, María-Ángeles Serrera-Figallo

**Affiliations:** 1 Department of Oral Surgery, Faculty of Dentistry, University of Seville, Seville, Spain; 2 Oral and Maxillofacial Unit, Virgen del Rocio Hospital, Seville, Spain. Oral Surgery Department, Faculty of Dentistry, University of Seville, Seville, Spain

## Abstract

**Background:**

The insertion torque of dental implants will depend on a combination of different factors such as bone density, the design of the implant and the drilling protocol used. However, it is not clear how the interaction of these factors affects the final insertion torque and which drilling protocol should be used in each clinical situation. The aim of this work is to analyse the influence of bone density, implant diameter and implant length on the insertion torque using different drilling protocols.

**Material and Methods:**

An experimental study was carried out in which the maximum insertion torque was measured, in standardised polyurethane blocks (Sawbones Europe AB) of four densities, for M12 Oxtein dental implants (Oxtein, Spain) with diameters of 3.5, 4.0, 4.5 and 5mm, and lengths of 8.5mm, 11.5mm and 14.5mm. All these measurements were carried out following four drilling protocols, a standard protocol, adding a bone tap, cortical drill or conical drill. In this way, a total of 576 samples were obtained. For the statistical analysis, the Table of confidence intervals, mean, standard deviation and covariance was carried out, in total and grouped by the parameters used.

**Results:**

The insertion torque for D1 bone obtained very high levels, reaching 77 6.95 N/cm, these values improved with the use of conical drills. In D2 bone, the mean torque obtained was 37.89± 13.70N/cm, with values within the standard. In D3 and D4 bone significantly low torques were obtained with values of 14.97± 4.40N/cm and 9.88± 4.16N/cm (*p*>0.001) respectively.

**Conclusions:**

In D1 bone, conical drills must be incorporated in drilling to avoid excessive torque, while in D3 and D4 bone, these would be contraindicated, as they drastically decrease the insertion torque, which may compromise the treatment.

** Key words:**Dental implant, torque, drilling protocols, bone density.

## Introduction

Since the beginnings of implantology, the importance of surgical drilling protocols in the long-term success of implants has been highlighted (1). The scientific evidence shows how the degree of primary stability obtained in the placement of implants depends on various factors, such as bone density (2), the characteristics of the implant (3,4) and the surgical technique (5). Several studies (6,7) have demonstrated that bone quality and the macrogeometric characteristics of the implant are factors which have the greatest influence on primary stability. Authors such as Möhlhenrich *et al* (8) determined that the diameter of the implant has a greater influence on primary stability than length and recommended selecting implants with different geometry in cases of low bone density to improve the primary stability obtained.

During planning of the surgery, the professional selects a specific drilling protocol based on the density and quality of the bone. One of the usual strategies for improving anchoring in cases of low bone density is infra-drilling (9,10). In the study by Coelho *et al*, the insertion torque was analysed in implants with different drilling protocols, showing that there is an inversely proportional relationship between the insertion torque and the preparation (infra-drilling, standard drilling or over-drilling) of the implant site (11). However, high torque does not always guarantee positive clinical results. Excessive tension or stress during the insertion of the implant may cause bone resorption (12).

In a multicentre retrospective study, Toia *et al* (13) analysed marginal bone loss in relation with the drilling protocol, bone density and insertion torque in patients treated with delayed implants and conventional loading. They found that cases with greater loss of crestal bone corresponded with those with greater bone density, insertion torque and those where surgical infra-drilling protocols had been followed.

However, recent systematic reviews (14-16) found no relationship between insertion torques, whether high or low, and the survival rate or marginal bone loss of implants.

Today, there is no consensus on the ideal insertion torque in each clinical situation. Although it is widely known that bone density, the drilling protocol, and the diameter and length of implants affect the insertion torque of our implants, it has not been studied how all these factors interact with each other and may alter the final torque, which is crucial in the long-term success of our rehabilitations on implants.

The aim of our study is to analyse the influence of bone density and the diameter and length of implants on the insertion torque using different drilling protocols.

## Material and Methods

A multifactorial experimental study was carried out, in which the maximum torque obtained under different conditions was measured. One type of implant with four diameters, three lengths, four bone densities (D1, D2, D3, D4) and four different scenarios corresponding to different surgical protocols have been compared. For each one of these factors, three samples were carried out, obtaining a total of 576 samples.

- Preparation in bone blocks

As a substitute, Sawbones Europe AB standardised bone blocks were used, made of urethanes, epoxy resins and structural fillings of four densities (17,18).

**Figure 1:**
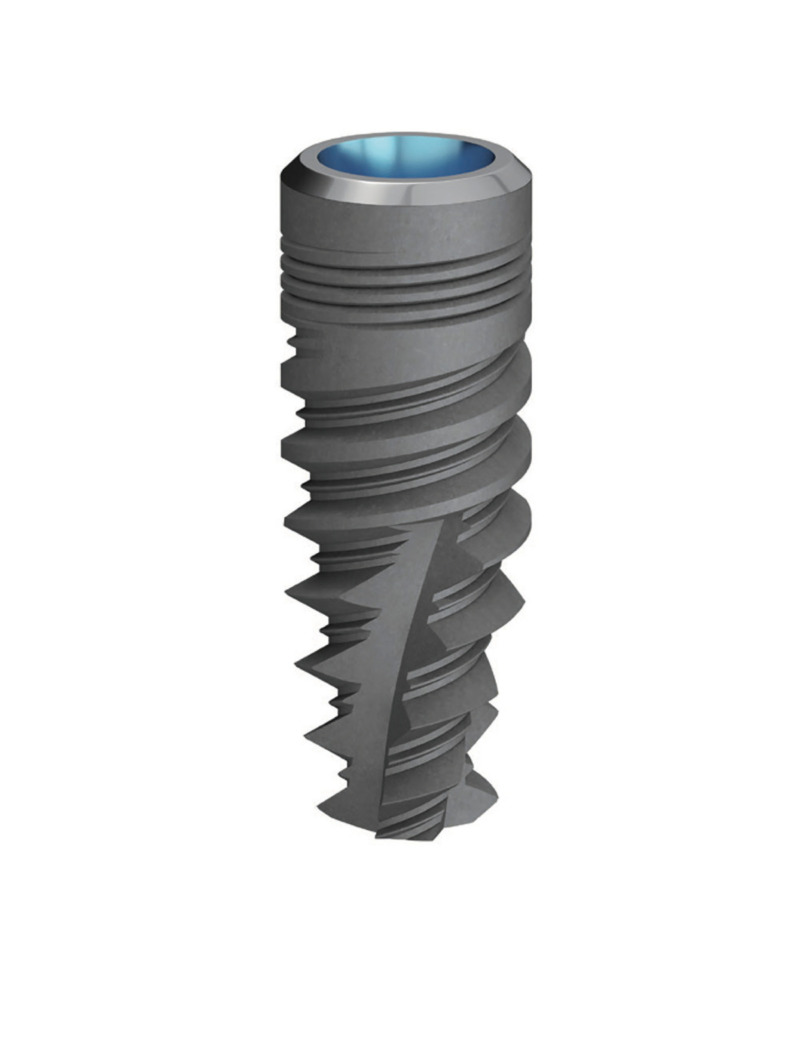
Oxtein M12 Implant (Oxtein, Madrid, Spain).

The implants used were Oxtein M12 (Oxtein, Madrid, Spain) with diameters of 3.5mm, 4.0mm, 4.5mm and 5.0mm and lengths of 8.5mm, 11.5mm and 14.5mm. Oxtein M12 is an internal connection implant with platform switching design, coronal microthreads, double "u" threads in the middle third and "v" threads in the apical area. It also has a self-tapping conical morphology and an atraumatic apex (Fig. 1).

The density of blocks D1, D2, D3 and D4 are respectively equivalent to:

Bone type I: Mostly cortical bone. Corresponds to Lekholm and Zarb type I bone.

Bone type II: Cortical bone 3-4mm thick surrounding a dense cancellous bone. Corresponds to Lekholm and Zarb type II bone.

Bone type III: Cortical bone 2mm thick surrounding a dense cancellous bone. Corresponds to Lekholm and Zarb type III bone.

Bone type IV: Mainly cancellous bone. Corresponds to Lekholm and Zarb type IV bone.

- Surgical drilling protocols:

For the preparation of the osteotomy for the implant, four drilling protocols were followed.

**Figure 2:**
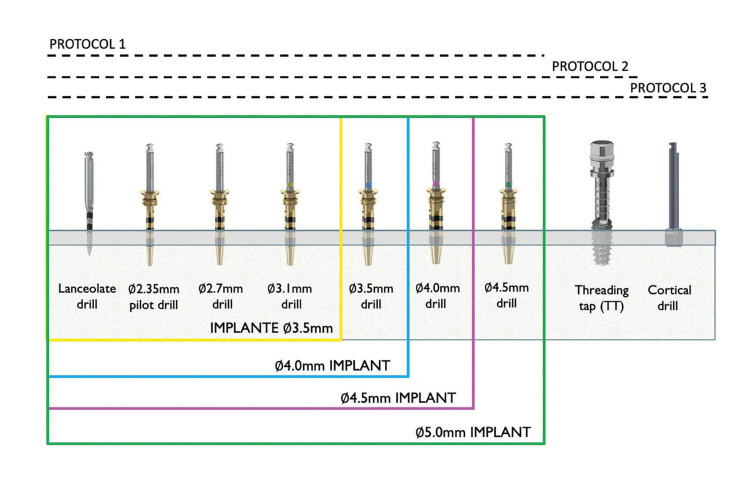
Drilling protocols.

Drilling protocol 1: Standard drilling. Drilling was started with a lanceolate drill and continued with a 2.35mm pilot drill, 2.7mm drill and 3.1mm drill in 3.5mm diameter implants. In 4.0mm implants, it was completed with the 3.5mm drill, in 4.5mm implants with the 4.0mm drill and in 5.0mm diameter implants with the 4.5mm drill (Fig. 2).

Drilling protocol 2: This protocol also incorporated the use of the threading tap (TT) corresponding to the diameter of the implant into to the standard drilling (Fig. 2).

Drilling protocol 3: Standard drilling is supplemented with the threading tap (TT) and a cortical drill (CD) (Fig. 2).

Drilling protocol 4: Standard drilling is followed by a conical drill corresponding to the diameter of the implant.

- Torque measurement

After the osteotomy, following the different drilling protocols, a manual insertion of the implant was carried out until reaching the juxta-osseous level. At this time, the measurement of the maximum insertion torque is carried out through digital ratchet (MSI Mark-10 model torque measurement device).

- Statistical analysis

Two statistical analyses of the data were carried out. Firstly, the Table of confidence intervals was created, also including the mean and standard deviation for the Torque value according to the different parameters used. The Student’s t-distribution was calculated for a sample, contrasting this sample with the Torque of 40N/cm. Additionally, the covariance was calculated as a general analysis for determining the significance of each variable, using diameter and length as factors and bone density and drilling protocol as covariables.

In the second analysis, the Table of confidence intervals was created for the Torque value based on the different parameters used, segmenting the information based on the bone density for the combinations of diameter and scenario and length and drilling protocol. In the same way, the confidence intervals were calculated based on the length and diameter of the implant. The latter data, as well as the whole study, is available to readers on request. In the presentation of the Tables, the statistical significance is indicated with the usual format (*p*<0.05; *p*<0.01; *p*<0.001, *p*<0,0001 and *p*<0.00001).

## Results

40N/cm (Student’s t-distribution) of insertion torque was taken as a reference value.

- Insertion torque and bone density

Type 1 bone (D1)

For shorter implants, those of 8.5mm, values within the standard were obtained in all protocols (Table 1). The 11.5 and 14.5mm implants obtained normal values only in drilling protocol 4 (41.75 9.79 N/cm and 40.58 13.71N/cm respectively); in the other protocols, without using a conical drill, the insertion torque was high with statistically significant results.

In regard to the diameter, confidence intervals were obtained in 3.5mm implants in drilling protocols 1, 2 and 3 (48.00 17.18 N/cm; 49.11 22.42 N/cm and 49.06 18.33 N/cm) while in protocol 4 the torque was lower (29.89 7.21 N/cm; *p*>0.01). For 4.0mm diameters, the use of the conical drill allowed normal values (38.44 3.243 N/cm). All 4.5mm diameter implants obtained insertion torque above the confidence interval. However, those of 5.0mm obtained values within the standard in drilling protocols 3 and 4 (49.06 13.46N/cm and 35.83 8.40 N/cm) (Table 1).

**Table 1:**
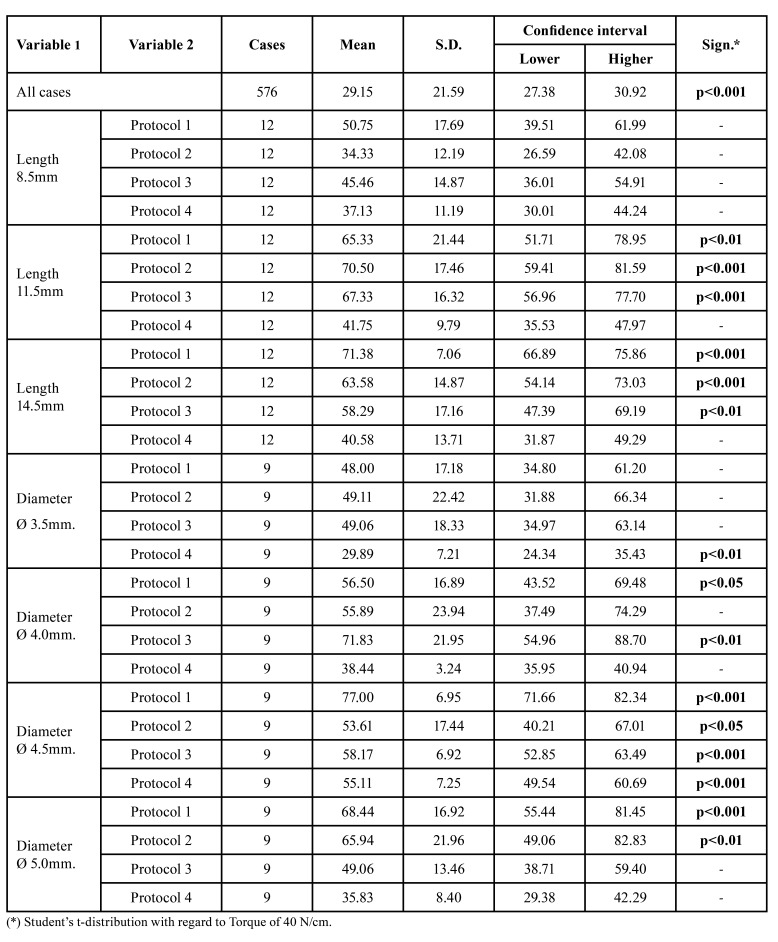
Confidence interval for Bone density 1.

Type 2 bone (D2)

The 8.5 and 11.5mm implants mostly obtained values within the standard, while those 14.5mm long had lower insertion torque with 29.63 4.73 N/cm in drilling protocol 4 (Table 2). The insertion torque was significantly low for 3.5mm diameter implants with all protocols. Additionally, the 4.0mm, 4.5mm and 5.0mm diameter implants had favourable results with values within the confidence interval.

**Table 2:**
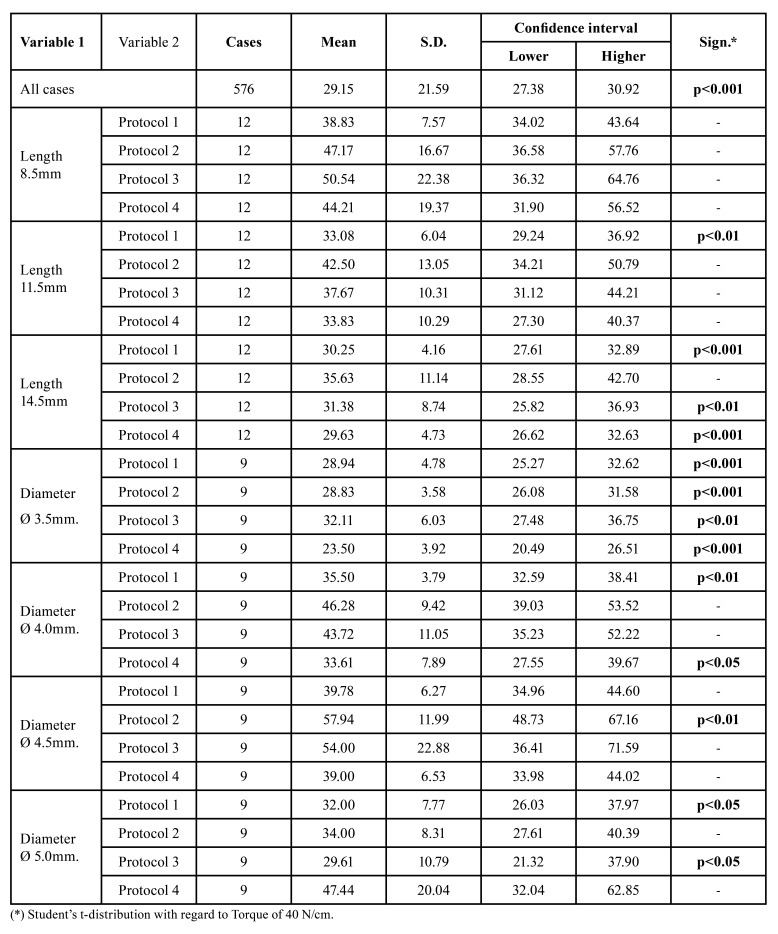
Confidence interval for Bone density 2.

Type 3 bone (D3)

In all drilling protocols, the values obtained were low, with statistically significant results (*p*>0.001). The lowest insertion torque, in type 3 bone, was the 3.5mm diameter implant in drilling protocol 4 (10.89 4.14 N/cm; *p*>0.001) (Table 3).

**Table 3:**
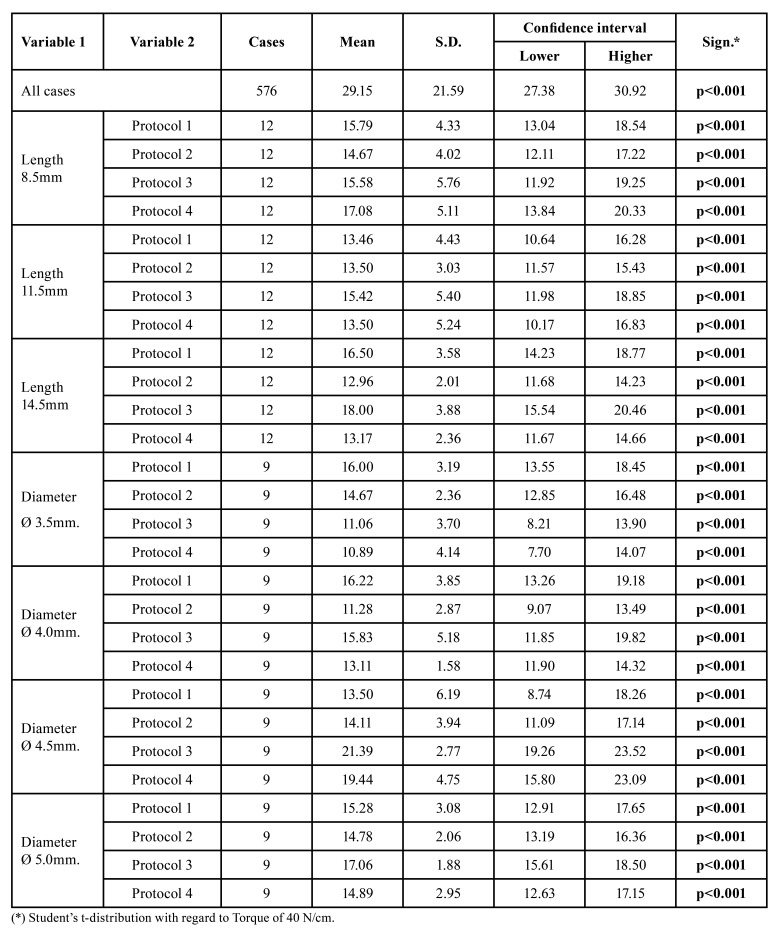
Confidence interval for Bone density 3.

Type 4 bone (D4)

In the same way, the results obtained in type 4 bone (D4) were significantly low, not reaching values within the standard using any of the drilling protocols, regardless of the length or diameter of the implant. In drilling protocol 4, adding the corresponding conical drill, the insertion torque reduced to 4.33 1.44 N/cm (*p*>0.001) in 5.0mm diameter implants (Table 4).

**Table 4:**
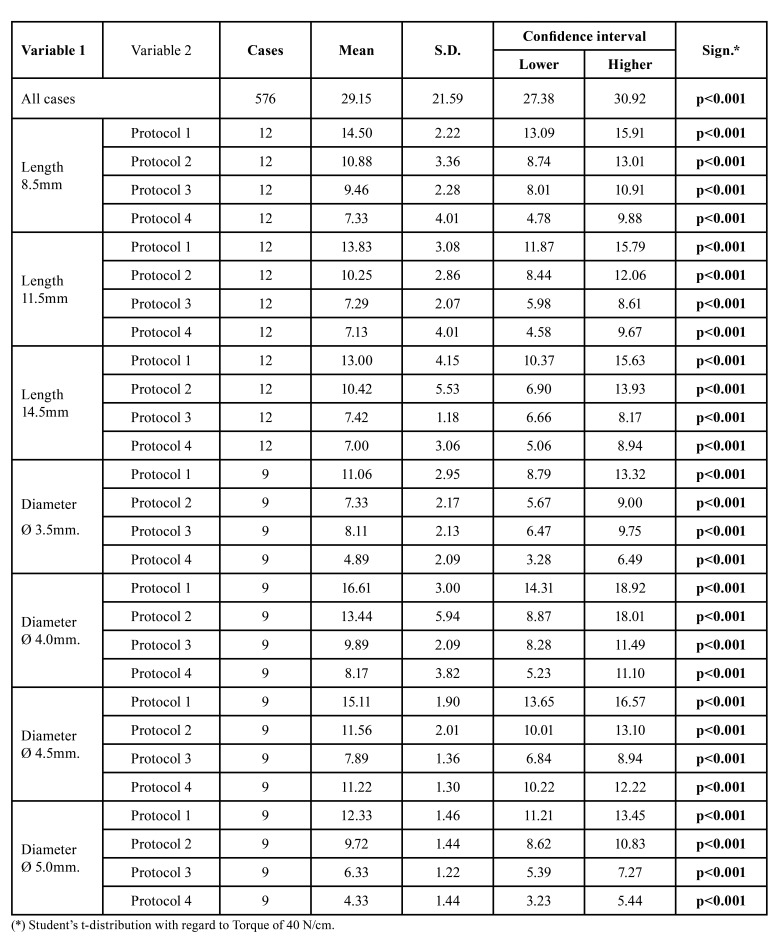
Confidence interval for Bone density 4.

- Insertion torque and length of the implant.

In the confidence analysis, for implants 8.5mm long, those 3.5mm in diameter obtained values below the confidence interval with torque of 24.549.81 N/cm, 19.96 9.02N/cm, 18.46 8.90N/cm, 15.54 7.69N/cm (*p*>0.001) in drilling protocols 1, 2, 3 and 4 respectively. Notably, for 4.0mm diameter implants, values within the confidence interval were only obtained in protocol 3, with torque of 30.33 19.72N/cm (*p*>0.001), the values being lower in the other protocols. For 4.5mm diameter implants, torque within the standard was obtained in all scenarios. Likewise, those 5.0mm in diameter obtained values within the confidence interval or very near to it, without significant values.

The 11.5mm long implants obtained values within the confidence interval for the majority of diameters and protocols. The exceptions, in which the torque obtained was less, were 3.5mm diameter implants in protocol 1 (23.08 14.42N/cm; *p*>0.01), 3.5mm diameter implants in protocol 4 (16.92 12.88N/cm; *p*>0.001), 4.0mm diameter implants in protocol 4 (22.04 12.25N/cm; *p*>0.001) and 5.0mm diameter implants in protocol 4 (26.79 19.85N/cm; *p*>0.05).

14.5mm long implants also mostly obtained values within the confidence interval. Lower insertion torque was obtained by 3.5mm diameter implants in protocol 2 (25.46 7.22, 16N/cm; *p*>0.05) and scenario 4 (19.42 12.32N/cm; *p*>0.001), 4.0mm diameter implants in protocol 4 (22.63 14.34N/cm; *p*>0.01) and 5.0mm diameter implants in protocol 2 (25.83 18.42N/cm; *p*>0.05), protocol 3 (20.04 12.00N/cm; *p*>0.001) and protocol 4 (18.75 10.70N/cm; *p*>0.001).

## Discussion

There are multiple factors which determine the final insertion torque of an implant. Many works have analysed how each one of these factors affect it separately; however, they must not be considered individually. Our study has the objective of evaluating the combined influence of bone density, the drilling protocol used, and the length and diameter of the implant on the final torque obtained.

In the attempt to find the ideal torque, many authors have analysed the influence of high torques compared with low torques. Duyck *et al* observed that a high insertion torque of over 45N increased the risk of bone microfractures with a greater bone resorption response at a molecular and cellular level, which led to a significant loss of bone stability in the first three weeks of healing (19). In a histological study, Cohen *et al* observed that after three weeks, the implants inserted with an infra-drilling protocol had large areas of bone resorption in combination with areas of bone formation in contact with the surface of the implant, while those placed with standard drilling had extensive areas of formation of new bone without signs of bone resorption (20).

Today, there is still dispute over how high insertion torque affects the stability of peri-implant tissues. On the one hand, randomised clinical studies such as those by Marconcini *et al* showed that marginal bone loss and soft tissue recession was statistically greater in implants placed with high torque (50N) compared with those placed with normal torque (<50N), thus noting the importance of bone density in planning implant surgery (21). However, several recent systematic reviews have analysed marginal bone resorption in implants placed with high torque compared with normal or low torque without finding statistically significant differences connecting these two factors (15,16).

Likewise, a very low torque may also affect our treatments. From the beginning of the development of implantology, the need for and importance of obtaining adequate primary stability has been described (1). The first works suggested that low initial torque during the placement of implants was associated with fibrointegration of the implant as a consequence of micromovements (22).

The final insertion torque plays a very important role in the case of immediate loading. In a recent systematic review and meta-analysis, Atieh *et al* found greater failure in implants placed with less than 50N of insertion torque compared with those placed with 50N, while there was no difference in survival with regard to insertion torque in implants with delayed loading (23).

The final insertion torque will depend on the interaction of different factors. In our work we have analysed how the combination of bone density, length and diameter of the implant and drilling protocol can affect the final torque and which of these factors will affect it most.

Several studies have described modifications to drilling protocols, with infra-drilling, to successfully obtain primary stability in low density bone with both delayed loading (24,25) and immediate loading (26). In their work, Anitua *et al* (27), sought to create a protocol for infra-drilling in implant surgery, based on bone density, to obtain adequate primary stability. To do so, they modified the drilling protocol, reducing it by 0.2, 0.4, 0.7, 1.0 and 1.2mm in bone type I, II, III, IV and V respectively, obtaining good insertion torque in all cases except bone type V (bone type IV in the Lekholm and Zarb classification) where an insertion torque of 5N/cm was obtained.

However, Farronato *et al*, in a quite similar study, evaluated the influence of bone quality, drilling protocol, implant length and diameter on insertion torque and determined that underpreparing protocols could lead to excessive insertion torque. To avoid undesirable bone compression they recommended dense bone protocol whenever high values of torque are expected (28).

Our work has shown that in D1 bone, with standard drilling, the insertion torque obtained is significantly high, which may compromise bone viability due to microfractures and overheating. 65.33 21.44 N/cm was obtained in implants 11.5mm long, 71.38 7.06 N/cm in implants 14.5mm long, 77 6.95 N/cm in implants 4.5mm in diameter and 68.44 16.92 N/cm in implants 5.0mm in diameter (Table 1).

These values compel us to modify drilling protocols to obtain an adequate insertion torque in very cortical and high-density bones, type D1 bone. In our study this was achieved by using conical drills, although for narrower implants, the use of conical drills may compromise primary stability.

Conversely, in type 4 bone (D4) we will obtain a low average torque (9.88 4.16 N/cm) regardless of the drilling protocol or length or diameter of the implant. Additionally, we must take into account that the use of conical drills will drastically reduce our insertion torque, even with values below 5N/cm (4.89 2.09 N/cm in 3.5mm diameter implants and 4.33 1.44N/cm in 5.0mm diameter implants). These results are consistent with those of Anitua *et al* (28), which shows us the importance of establishing infra-drilling protocols in type IV cancellous bone, completely omitting the use of additional drills such as bone taps, cortical drills or conical drills.

Our results show that the most critical factor for obtaining a high insertion torque (>40N) is bone density. Other factors such as the length or diameter of the implant or the drilling protocol will not in themselves have a great impact in obtaining sufficient primary stability, although their combination may seriously compromise it.

It is important to mention that one of the limitations of our *in vitro* study is that it only considers mechanical aspects. Therefore, we were not able to analyse the osseointegration of implants based on the torque obtained or evaluate its long-term impact.

The insertion torque of dental implants will depend on the interaction of different factors such as bone density, the macroscopic design of the implant and the drilling protocol.

Our drilling protocols must be adapted to the bone density of the area to rehabilitate.

1. In bone type 1, incorporating conical drills into the drilling protocol is crucial for avoiding excessive insertion torques which compromise the vascularisation and viability of dental implants.

2. In bone type 2, we obtain normal insertion torques and the drilling protocol will not be as relevant. An exception are narrow implants, where we find low values, and where additional drills must be avoided.

3. In bone type 3 and type 4, the insertion torque will be very low. The use of additional drills such as the bone tap, cortical drills or conical drills are advised against and may fully compromise the success of the treatment. In these cases, infra-drilling should be considered.
